# NE52-QQ57 attenuates post-ischemia protein phosphorylation in the mouse brain

**DOI:** 10.64898/2026.10.09.754558

**Published:** 2026-10-12

**Authors:** Shambhu Joshi, Youfang Zhou, Xiang-ming Zha

## Abstract

**Introduction:**

Cerebral ischemia induces a reduction in brain pH. GPR4, a proton-sensing G protein-coupled receptor is highly expressed in vascular endothelial cells. Pharmacological inhibition of GPR4 with NE52-QQ57 (NEQQ) reduces ischemia-induced blood-brain barrier (BBB) disruption and brain injury. To facilitate targeting GPR4 in vivo, it is important to better define the effect of NEQQ on post-ischemia signaling and the pharmacokinetics of NEQQ *in vivo* .

**Methods:**

We performed biochemical analysis following transient middle cerebral artery occlusion (tMCAO). We used Liquid Chromatography-Mass spectrometry (LC-MS/MS) to quantify the concentration of NEQQ in serum, brain, and peripheral tissues.

**Results:**

NEQQ administration decreased the phosphorylation of PKA substrates, PKC substrates, ERK1/2, and CREB. LC-MS/MS analysis detected NEQQ in both ipsilateral and contralateral hemisphere of the brain at 5 h and 24 h after reperfusion, demonstrating that NEQQ crosses the BBB. In peripheral tissues, NEQQ exhibited highest level in kidney and lowest in the heart. In sham controls, average serum concentrations were ∼4 and 0.9 µg/g at 5 and 24 h, respectively. Following tMCAO, ipsilateral brain exhibited higher levels as compared to the contralateral side at 5 h while the difference diminished at 24 h.

**Conclusions:**

NEQQ attenuated the ischemia-induced activation of multiple signaling pathways. NEQQ exhibited broad distribution in peripheral organs, and its brain level was sufficient for GPR4 inhibition at 24 h after injection. These findings support the use of NEQQ as a pharmacological inhibitor of GPR4-mediated signaling following ischemic stroke.

